# Draft Genome Sequence of *Enterococcus faecalis* Strain PF3, Isolated from Adélie Penguin Feces from Antarctica

**DOI:** 10.1128/genomeA.01209-13

**Published:** 2014-01-23

**Authors:** Robert J. Spence, Ana Pavasovic, James J. Smith, Peter J. Prentis

**Affiliations:** a Science and Engineering Faculty, Queensland University of Technology, Brisbane, Queensland, Australia; b Faculty of Health, Queensland University of Technology, Brisbane, Queensland, Australia

## Abstract

*Enterococcus faecalis* is one of the leading causes of nosocomial infections and is a common commensal organism in humans and other animals. In this study, we report a draft genome sequence for the *E. faecalis* strain PF3, isolated from Adélie penguin feces collected from Warriner Island, Antarctica.

## GENOME ANNOUNCEMENT

*Enterococcus* spp. are Gram-positive bacteria commonly found under a wide range of different environmental conditions (1). Within this genus, *Enterococcus faecalis* is a common commensal organism found in the gastrointestinal tract of warm-blooded animals (2). This species has been isolated from a range of different organisms, including mammals, birds, food industry animals, insects, and plants (3). Here, we completed a draft genome sequence for *E. faecalis* strain PF3, isolated from Adélie penguin (*Pygoscelis adeliae*) feces collected on Warriner Island, Antarctica.

The genome of *E. faecalis* PF3 was sequenced on an Ion Proton PGM using a P1 chip with 200-bp chemistry. The raw data comprise 921 megabases with 8,723,410 reads averaging 105.6 bp. *De novo* assembly was performed using CLC Genomics Workbench 6.02, producing 401 contigs, with an N_50_ of 59,627 bp and with the largest contig being 201,859 bp. The draft genome that resulted from this assembly is 3,215,729 bp long with a G+C content of 37.5%. xBASE annotation (4–9) detected a total of 3,387 genes, of which 3,338 are coding sequences (CDSs), 45 are tRNA genes, and 4 are rRNA genes.

Progressive Mauve (10) was used to align *E. faecalis* PF3 to other *E. faecalis* reference genomes. Pairwise alignments determined that the PF3 strain shows the greatest homology to the widespread commensal *E. faecalis* OG1RF strain. The draft genome contains a pathogenicity island, multiple plasmid sequences, and three clusters of regularly interspaced short palindromic repeat (CRISPR)-associated genes.

### Nucleotide sequence accession numbers.

This whole-genome shotgun project has been deposited at DDBJ/EMBL/GenBank under the accession no. AZIA00000000. The version described in this paper is version AZIA01000000.
